# Meta-QTL analysis and identification of candidate genes for multiple-traits associated with spot blotch resistance in bread wheat

**DOI:** 10.1038/s41598-024-63924-w

**Published:** 2024-06-07

**Authors:** Neeraj Kumar Vasistha, Vaishali Sharma, Sahadev Singh, Ramandeep Kaur, Anuj Kumar, Vikas Kumar Ravat, Rahul Kumar, Pushpendra K. Gupta

**Affiliations:** 1 Department of Genetics and Plant Breeding, Rajiv Gandhi University, Rono Hills, Itanagar, India; 2 Department of Genetics-Plant Breeding and Biotechnology, Dr K. S. Gill, Akal College of Agriculture, Eternal University, Baru Sahib, Sirmour, India; 3 Molecular Biology Laboratory, Department of Genetics and Plant Breeding, Ch. Charan Singh University, Meerut, India; 4 Meerut Institute of Technology, NH-58 Baral Partapur Bypass Road, Meerut, India; 5 Department of Plant Pathology, Rajiv Gandhi University, Rono Hills, Itanagar, India; 6 Murdoch’s Centre for Crop and Food Innovation, Murdoch University, Murdoch, WA Australia; 7 Borlaug Institute for South Asia (BISA), National Agricultural Science Complex (NASC), Dev Prakash Shastri (DPS) Marg, New Delhi, India

**Keywords:** Wheat, *Bipolaris sorokiniana*, Meta-QTL, Spot blotch, Candidate genes, Biotechnology, Computational biology and bioinformatics, Genetics, Plant sciences

## Abstract

In bread wheat, a literature search gave 228 QTLs for six traits, including resistance against spot blotch and the following five other related traits: (i) stay green; (ii) flag leaf senescence; (iii) green leaf area duration; (iv) green leaf area of the main stem; and (v) black point resistance. These QTLs were used for metaQTL (MQTL) analysis. For this purpose, a consensus map with 72,788 markers was prepared; 69 of the above 228 QTLs, which were suitable for MQTL analysis, were projected on the consensus map. This exercise resulted in the identification of 16 meta-QTLs (MQTLs) located on 11 chromosomes, with the PVE ranging from 5.4% (MQTL7) to 21.8% (MQTL5), and the confidence intervals ranging from 1.5 to 20.7 cM (except five MQTLs with a range of 36.1–57.8 cM). The number of QTLs associated with individual MQTLs ranged from a maximum of 17 in MQTL3 to 8 each in MQTL5 and MQTL8 and 5 each in MQTL7 and MQTL14. The 16 MQTLs, included 12 multi-trait MQTLs; one of the MQTL also overlapped a genomic region carrying the major spot blotch resistance gene *Sb1*. Of the total 16 MQTLs, 12 MQTLs were also validated through marker-trait associations that were available from earlier genome-wide association studies. The genomic regions associated with MQTLs were also used for the identification of candidate genes (CGs) and led to the identification of 516 CGs encoding 508 proteins; 411 of these proteins are known to be associated with resistance against several biotic stresses. In silico expression analysis of CGs using transcriptome data allowed the identification of 71 differentially expressed CGs, which were examined for further possible studies. The findings of the present study should facilitate fine-mapping and cloning of genes, enabling Marker Assisted Selection.

## Introduction

Wheat is an important cereal crop, cultivated worldwide for a variety of products, and is consumed as a regular diet world-wide by billions of people. It has also been described as ‘the King of Cereals’ because of the golden colour of its grain^1^. Global wheat production showed a marginal increase from 778 million metric tons (MMT) in the year 2022, to 779.6 MMT in 2023 to an estimated all time high of 797 MMT in the current year 2024 (https://www.ers.usda.gov/webdocs/outlooks/104470/whs-22h.pdf?v=8189). India makes a major contribution to this global production, being only second to China in its annual production. It is cultivated in a variety of soils and climates. As a result, major losses in grain yield in wheat are caused due to a variety of biotic and abiotic stresses; the biotic stresses included several diseases, including spot blotch (SB), which causes a loss of 15–25%, especially in warm and humid areas of countries including Bangladesh, Nepal, Brazil, India, and Zambia^2^. The disease also affects the quality, texture, and colour of harvested wheat grains^3^.

Resistance against individual diseases in all major crops, including wheat, has largely been treated as a qualitative trait. Therefore, R genes for resistance have been identified and utilized for conventional methods of plant breeding. Spot blotch is no exception to this trend, so that four major genes, namely *Sb1* to *Sb4* following a gene-for-gene (GFG) relationship, are now known and have been largely used for resistance breeding; more recently, the *Tsn1*-ToxA system for sensitivity to this disease following an inverse gene-for-gene (IGFG) relationship has also been discovered^4–8^. During the last two decades, however, resistance against most diseases, including spot blotch, has been treated as a quantitative trait for genetic studies. These relatively recent studies included linkage-based interval mapping for the identification of Quantitative Trait Loci (QTLs) and LD-based GWA (Linkage Disequilibrium-based Genome Wide Association) studies for the identification of MTAs. As a result, a large number of QTLs/MTAs have been identified for each individual disease, including spot blotch. However, relatively few individual QTLs or MTAs have been utilized successfully for MAS, leading to the development of resistant cultivars. This is attributed to the non-availability of robust QTLs/MTAs for diverse genetic backgrounds and environments. MQTL analysis is a technique that provides a solution to this problem, so that a large number of MQTL studies for disease resistance in wheat have recently been conducted, either for resistance against individual diseases or for multiple disease resistance (MDR). Following are some examples of individual diseases: stem rust^9^, fusarium head blight^10–13^, tan spot^14^, stripe rust^15,16^, leaf rust^17,18^. Similarly, examples of MQTL analysis for MDR include two of our own studies for resistance against five diseases in one study^19^ and resistance against all three rusts in another study^20^.

For spot blotch in wheat, more than a dozen studies involving interval mapping have so far been conducted^2^. However, for the reasons mentioned above, only sparingly QTLs have been recommended and used for the transfer of spot blotch resistance using MAS^21^. The use of MAS for breeding resistant cultivars will, however, be facilitated if robust MQTLs with high phenotypic variation explained (PVE) and narrow confidence interval (CI) are available. Keeping this in view, the present study was planned, where QTLs not only for SBR, but also those for some other associated traits were utilized for the development of MQTLs. These other traits included the following traits: (i) stay green (SG); (ii) flag leaf senescence (FLS); (iii) green leaf area duration (GLAD); (iv) green leaf area of the main stem (GLMS); and (v) black point resistance (BPR). These other traits were found to be associated with spot blotch in several QTL interval studies^22–28^. The present study is the first study involving MQTL analysis for spot blotch resistance and associated traits for the development of robust markers to be used for MAS. Candidate genes (CGs) associated with genomic regions occupied by MQTLs were also identified for a better understanding of the genetic basis of spot blotch resistance in wheat.

## Results

### Bibliography search

**Figure 1:**
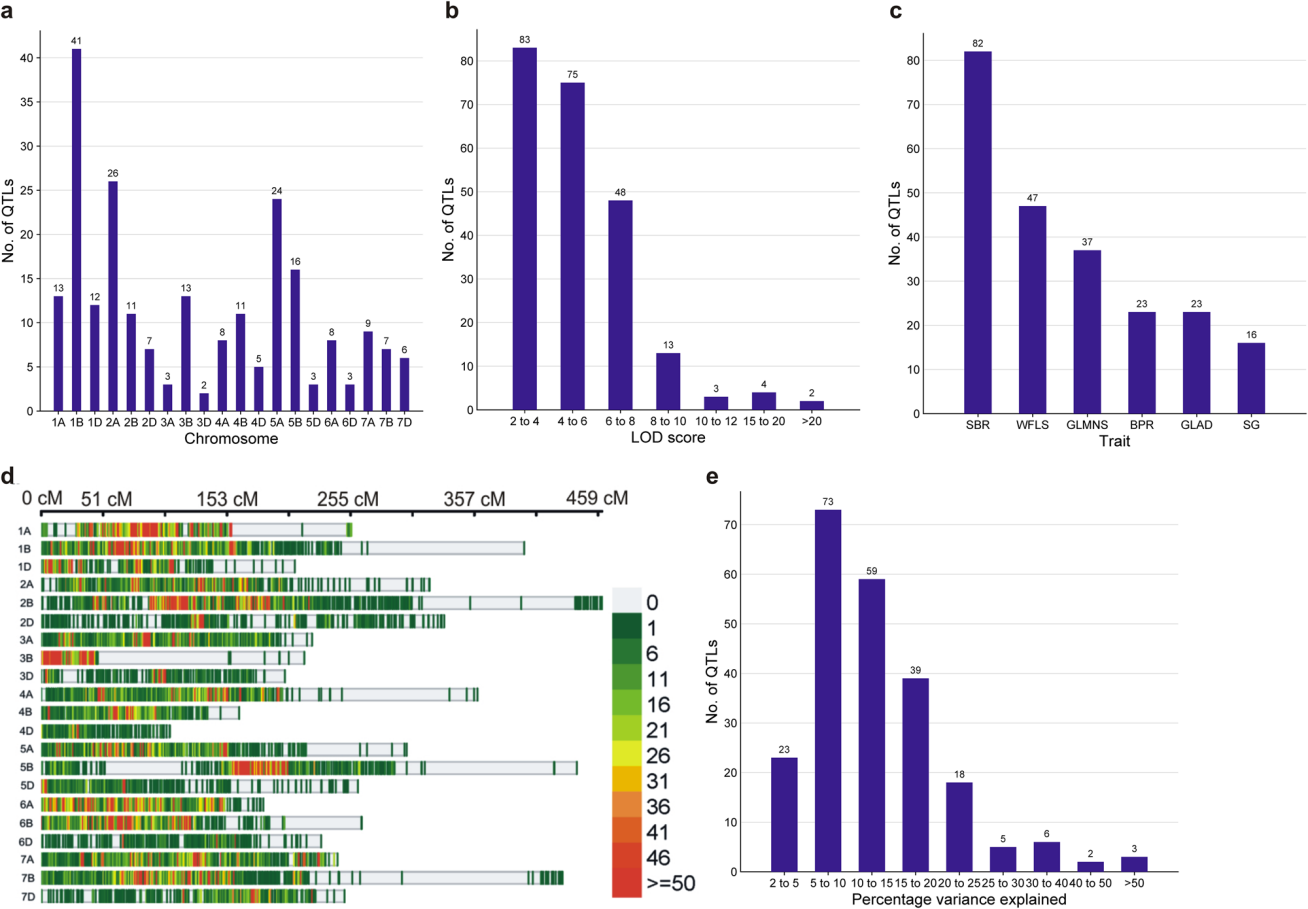
The data utilised in the MQTL analysis. (**a**) frequencies of QTLs used to identify MQTLs on each of the 20 wheat chromosomes (except 6B); (**b**) frequencies of QTLs with different LOD scores; (**c**) frequencies of QTLs for different traits; (**d**) density of molecular markers on consensus map involving 21 wheat chromosomes; (**e**) frequencies of QTLs with different PVE% values.

A literature search involving QTL interval mapping for spot blotch and related traits was undertaken, which resulted in the identification of 228 QTLs from 28 studies involving 24 mapping populations (Supplementary Table S1). These QTLs were located on 20 different wheat chromosomes (except 6B; Fig. 1a). The maximum number of QTLs was available on chromosome 1B (41) followed by 2A (27) and 5A (23); the minimum number of only one QTL was available on 5D (Fig. 1a, b, c, e).

### Consensus map and QTL projection

**Figure 2:**
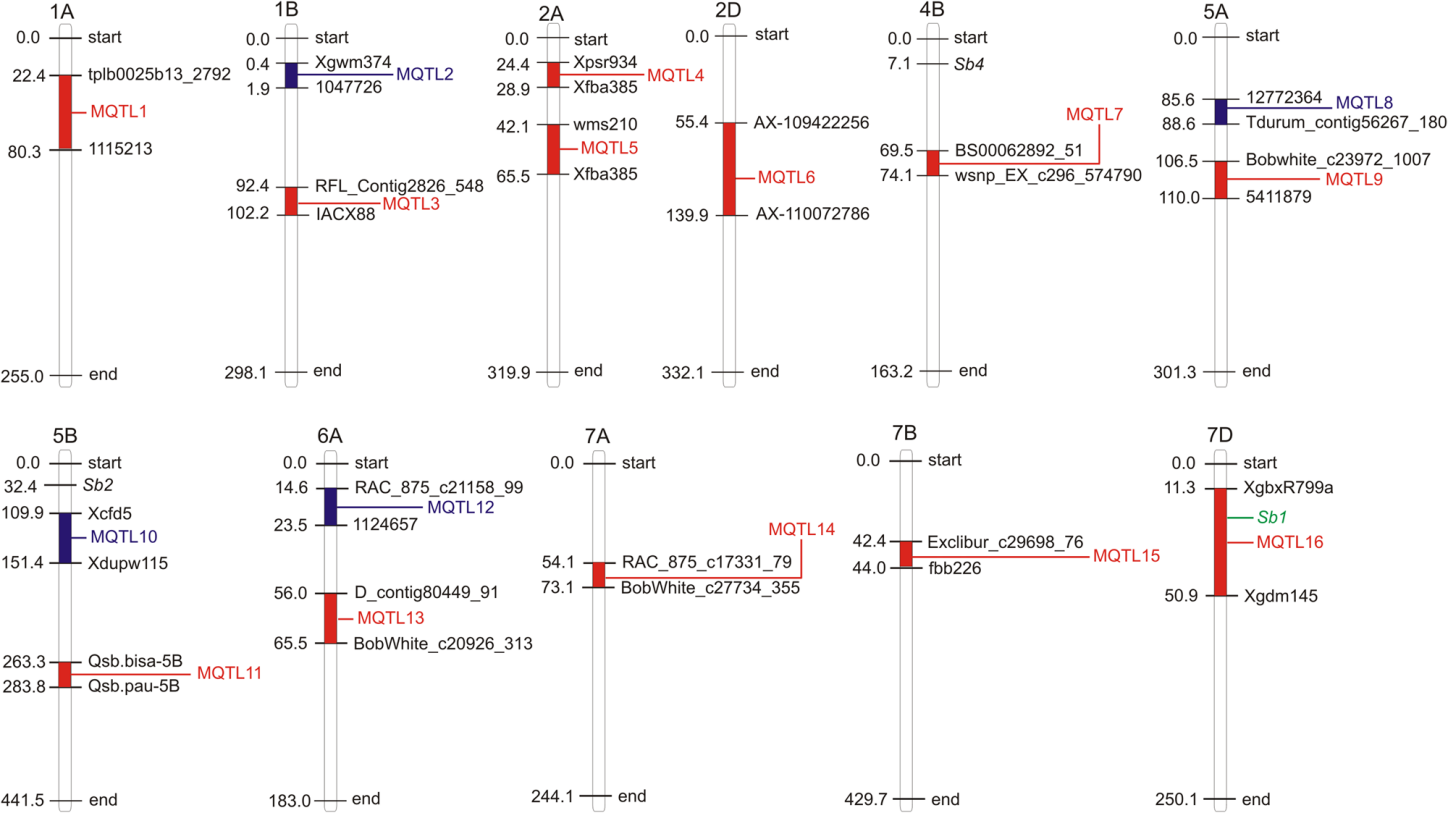
MQTLs are distributed on various wheat chromosomes, with MQTLs shown by blue and GWAS validated MQTLs indicated by red.

An integrated consensus map was prepared using markers that were utilized for interval mapping in all earlier studies. This map carried 72,788 marker loci (mainly SNPs, SSRs, and DArT markers; SNP = Single Nucleotide Polymorphism; SSR = Simple Sequence Repeat; DArT = Diversity Array Technology) (Fig. 1d, Supplementary Table S2). The number of markers on individual chromosomes ranged from a minimum of 591 (4D) to a maximum of 7,126 (1A). The genetic lengths of individual chromosomes ranged from 163.2 cM (4B) to 441.5 cM (5B) (Fig. 2) The minimum length between adjacent markers on a chromosome was 6.9 cM on chromosome 7D, and the maximum length (27.9 cM) on chromosome 1A (Supplementary Table S3). Only 84 of the above 228 QTLs could be used for projection; the remaining 144 QTLs did not have the complete information required for projection. Out of 84 projected QTLs, only 69 were useful (Table 1); the remaining 15 QTLs were singleton (each based on a single QTL) and therefore, were not utilised in downstream analysis. Among the 69 projected QTLs, the highest number of 23 QTLs were available for the trait GLAD (33.3%), followed by 20 (29.0%) for SBR and 19 (27.5%) for GLMS.

**Table 1:** Summary of the results of MQTL analysis for resistance to spot blotch disease in wheat.

MQTL-Chr/QTLs in MQTL region	Peak/CI (cM)	PVE%/(avg. LOD score)	Flanking markers	Associated traits with no. of QTLs	Study involved
MQTL1-1A/2	73.8/57.8	14.0 (5.4)	*tplb0025b13_2792-1115313*	SBR (1) GLMS (1)	Shi et al.^28^, Roy et al.^29^
MQTL2-1B/2	1.2/1.5	16.0 (3.4)	*Xgwm374-1047726*	SBR (2)	Zhu et al.^30^, Singh et al.^31^
MQTL3-1B/17	97.2/10.0	14.2 (6.9)	*RFL_Contig2826_548-IACX886*	SBR (2) GLAD (11) GLMS (4)	Wang et al.^24^, Shi et al.^28^, Pankaj et al.^32^, Ghatyari et al.^33^
MQTL4-2A/4	26.7/4.5	10.7(5.5)	*Xpsr934-Xfba385*	GLMS (4)	Shi et al.^28^
MQTL5-2A/8	52.3/2.4	21.8 (4.7)	*wms210-wmc474*	SBR (1) BPR (1) GLMS (4) GLAD (2)	Kumar et al.^22^, Shi et al.^28^, Gao et al.^34^
MQTL6-2D/3	85.5/8.3	16.0 (5.4)	*AX-109422256-AX-110072786*	SBR (2) BPR (1)	Kumar et al. ^22^, Pankaj et al.^32^, Gao et al.^34^
MQTL7-4B/5	71.8/4.6	5.4 (5.5)	*BS00062892_51-wsnp_Ex_c296_574790*	SBR (2) SG (1) GLMS (2)	Kumar et al.^23^, Shi et al. ^28^, Singh et al. ^31^, Ghatyari et al. ^33^
MQTL8-5A/8	86.8/3.6	13.6 (4.8)	*12772364-Tdurum_contig56267_180*	SBR (2) GLAD (5) GLMS (1)	Wang et al. ^24^, Shi et al.^28^, Zhu et al.^30^, Pankaj et al.^32^
MQTL9- 5A/2	108.3/3.5	18.0 (4.5)	*Bobwhite_c23972_1007-5411879*	GLAD (1) FLS (1)	Wang et al. ^24^, Shi et al. ^28^
MQTL10-5B/3	130.3/42.1	14.4 (6.1)	*Xcfd5-Xdupw115*	SG (1) GLAD (1) GLMS (1)	Wang et al. ^24^, Shi et al.^28^, Chrishtofer et al.^35^
MQTL11-5B/2	303.3/20.7	19.0 (3.3)	*Q.sb.bhu-5B-Q.sb.bisa-5B*	SBR (2)	Kumar et al.^22^, Roy et al. ^29^
MQTL12-6A/2	22.7/10.3	13.0 (5.8)	*RAC875_c21158_99-1124657*	SBR (1) GLMS (1)	Shi et al.^28^, Pankaj et al.^32^
MQTL13-6A/2	61.5/36.1	10.5 (5.2)	*D_contig80449_91-BobWhite_c20926_313*	SBR (1) FLS (1)	Wang et al. ^24^, Roy et al. ^29^
MQTL14-7A/5	63.6/19.1	10.8 (11.5)	*RAC875_c17331_79-BobWhite_c27734_355*	FLS (1) GLAD (3) GLMS (1)	Wang et al. ^24^, Shi et al. ^28^
MQTL15-7B/2	43.2/1.8	8.5 (3.8)	*Exclibur_c29698_76-fbb226*	SBR (2)	Kumar et al.^22^, Singh et al.^36^
MQTL16-7D/2	37.9/38.7	9.0 (3.2)	XgbxR799a-Xgdm145*	SBR (2)	Lillemo et al.^4^, Kumar et al.^22^

*Sb1 gene is located within the interval of markers.

### MQTL analysis

MQTL analysis gave 16 MQTLs distributed on 11 wheat chromosomes (Table 1). The number of QTL involved in individual MQTL ranged from a maximum of 17 QTLs in MQTL3, 8 each in MQTL5 and MQTL8 and 5 each in MQTL7 and MQTL14; the remaining MQTLs were each based on < 5 QTLs (Table 1). The number of traits controlled by individual MQTL ranged from 4 traits (SBR, GLAD, GLMS, and BPR) involved for MQTL5 to only single traits (SBR) in each of five MQTLs (MQTL2, MQTL4, MQTL11, MQTL15 and MQTL16) (Table 1). Out of the six traits, SBR was controlled by 12 (75%) out of 16 MQTLs, while GLMS was controlled by each of 9 MQTLs (56.2%); SG and BPR were each controlled by only 2 MQTLs (12.5%). The distribution of MQTLs on three sub-genomes also differed with 8 MQTLs on A sub-genome, 6 on B sub-genome and 2 on D sub-genome (Fig. 2). Similarly, chromosome-wise distribution of MQTLs ranged from a maximum of 2 each on five chromosomes, namely 1B, 2A, 5A, 5B and 6A and a minimum of 1 each on several other chromosomes (Fig. 2). Mean PVE % for individual MQTLs ranged from 5.4 to 21.8% (mean 13.5), the logarithm of odd (LOD) ranged from 3.2 to 11.5 (mean 5.3) and the CI for individual MQTLs ranged from minimum 1.5 cM (MQTL2) to a maximum on 57.8 cM (MQTL1) with a mean of 16.5 cM (Table 1). This amounted to 4.6 times reduction in the length of CI in original QTLs. The average length of CI on chromosomes 2A was reduced by 27.7 fold and that on chromosome 5A was reduced by 26.4 fold, followed by 12.7 and 8.4 fold reduction in MQTLs on chromosomes 1B and 4B. The spot blotch resistance gene, *Sb1*^4^ was associated with MQTL16 (Table 1, Fig. 2).

### Selection of major MQTLs

The above 16 MQTLs were also subjected to selection of the most important MQTLs using the following four criteria: (i) high frequency of QTLs (ranging from 4 to 17) associated with the corresponding individual MQTL; (ii) high PVE% (ranging from 10.7 to 21.8), (iii) high LOD score (ranging from 4.7 to 6.9) and (iv) relatively narrow CI (ranging from 2.4 cM to 10 cM). This exercise gave the following four major MQTLs: MQTL3, MQTL4, MQTL5, and MQTL8. These four major MQTLs were later also found to be associated with CGs that were particularly relevant to resistance against biotic stresses (Table 2).

**Table 2:** Candidate genes associated with each of four major MQTLs and their encoded proteins.

MQTL3
1. *TraesCS1B02G037100*: NAD(P)-binding domain, NAD(P)-binding domain superfamily, Sanguinarine reductase SARED1-like, MaoC-like dehydratase domain, HotDog domain superfamily, NB-ARC
2. *TraesCS1B02G036800*: P-loop containing nucleoside triphosphate hydrolase, Leucine-rich repeat domain superfamily, Winged helix-like DNA-binding domain superfamily, Disease resistance protein, plants
3. *TraesCS1B02G037000*: Multi antimicrobial extrusion protein, Multidrug and toxic compound extrusion family
4. *TraesCS1B02G035600*: Eukaryotic, NB-ARC, P-loop containing nucleoside triphosphate hydrolase, Winged helix-like DNA-binding domain superfamily, Virus X resistance protein-like, coiled-coil domain, Rx, N-terminal, Apoptotic protease-activating factors, helical domain, Disease resistance protein, plants
5. *TraesCS1B02G038100*: PGR5-like protein 1
6. *TraesCS1B02G036200*: P-loop containing nucleoside triphosphate hydrolase, Virus X resistance protein-like, coiled-coil domain, Mitochondrial import inner membrane translocase subunit Tim8/13, Rx, N-terminal
7. *TraesCS1B02G389000*: Splicing factor 3B subunit 5/RDS3 complex subunit 10
8. *TraesCS1B02G035800*: Glyoxalase/Bleomycin resistance protein/Dihydroxybiphenyl dioxygenase, Vicinal oxygen chelate (VOC) domain, 4-hydroxyphenylpyruvate dioxygenase, C-terminal, N-terminal
9. *TraesCS1B02G036600*: Protein BRANCHLESS TRICHOME-like, Dirigent protein
10. *TraesCS1B02G036100*: Proteinase inhibitor I13, potato inhibitor I superfamily, Calcium-dependent channel, 7TM region, putative phosphate, 10TM putative phosphate transporter, cytosolic domain, Calcium permeable stress-gated cation channel 1, N-terminal transmembrane domain
MQTL4
1. *TraesCS2A02G193300*: Interferon-related developmental regulator, N-terminal, Armadillo-like helical
2. *TraesCS2A02G193700*: Formin, FH2 domain
3. *TraesCS2A02G194200*: Myc-type, basic helix-loop-helix
4. *TraesCS2A02G194400*: Queuosine salvage protein family
MQTL5
1. *TraesCS2A02G012800*: A1 cistron-splicing factor, AAR2, N-terminal, C-terminal domain superfamily, N-terminal domain superfamily
2. *TraesCS2A02G013000:* Cytochrome P450, E-class, group IV, conserved site, conserved site, E-class, group I
3. *TraesCS2A02G013600:* Carbohydrate kinase PfkB, Ribokinase-like
4. *TraesCS2A02G014100:* Ribosome-inactivating protein, subdomain 1, Ribosome-inactivating protein superfamily, Muniscin C-terminal, Mu homology domain, AP-2 complex subunit mu, C-terminal superfamily
5. *TraesCS2A02G014400:* DNA-binding pseudobarrel domain superfamily
6. *TraesCS2A02G014800:* Chalcone/stilbene synthase, N-terminal, Polyketide synthase, type III
7. *TraesCS2A02G014900:* C-terminal, Thiolase-like, Cytochrome P450, E-class, group I, Cytochrome P450, conserved site, PAZ domain, Piwi domain
8. *TraesCS2A02G168900:* PAZ domain, Piwi domain, Ribonuclease H-like superfamily, Argonaute, linker 1 domain, Protein argonaute, Mid domain, N-terminal, Ribonuclease H superfamily
MQTL8
1. TraesCS5A02G487900: Sugar phosphate transporter domain
2. TraesCS5A02G488200: Trafficking protein particle complex subunit, Longin-like domain superfamily
3. TraesCS5A02G488700: RNA recognition motif domain, Zinc finger, CCCH-type, Nucleotide-binding alpha–beta plait domain superfamily, RNA-binding domain superfamily, Zinc finger, CCCH-type superfamily, Ist3-like, RNA recognition motif
4. TraesCS5A02G489300: Glycolipid transfer protein domain, Glycolipid transfer protein superfamily
5. TraesCS5A02G489400: Glycolipid transfer protein domain, Glycolipid transfer protein superfamily
6. TraesCS5A02G489800: Phosphoesterase, Alkaline-phosphatase-like, core domain superfamily, Phosphoesterase, Alkaline-phosphatase-like, core domain superfamily

### Comparing MQTLs with GWAS-MTAs

Effort was also made in the present study to validate MQTLs using GWAS-based MTAs for spot blotch and related traits. A literature search identified 606 MTAs for all the six traits from 22 GWA studies. The locations of these MTAs were compared with those of the above 16 MQTLs. The number of MTAs associated with individual MQTL ranged from 2 (MQTL16) to 28 (MQTL5) (Supplementary Table S4) and involved only three of the six traits, namely SBR, SG, and BPR (the number of MTAs for each MQTL differed; details are provided in Supplementary Table S4). Trait-wise individual MQTLs colocalized with MTAs included the following: (i) for SBR, 12 MQTL (MQTL1, MQTL3, MQTL4, MQTL5, MQT6, MQTL7, MQTL9, MQTL11, MQTL13, MQTL14, MQTL15, and MQTL16); (ii) for SG, 8 MQTLs (MQTL1, MQTL3, MQTL5, MQTL6, MQTL7, MQTL9, MQTL14, and MQTL15); and (iii) for BPR, 7 MQTLs (MQTL1, MQTL3, MQTL5, MQTL6, MQTL9, MQTL13, and MQTL14).

### Candidate genes

The genomic regions associated with the above 16 MQTLs carried 516 unique CGs, which encoded ~ 508 proteins; 411 of these proteins are already known to be associated with tolerance to a variety of biotic stresses, including resistance/sensitivity to spot blotch. (Supplementary Table S5).

**Figure 3:**
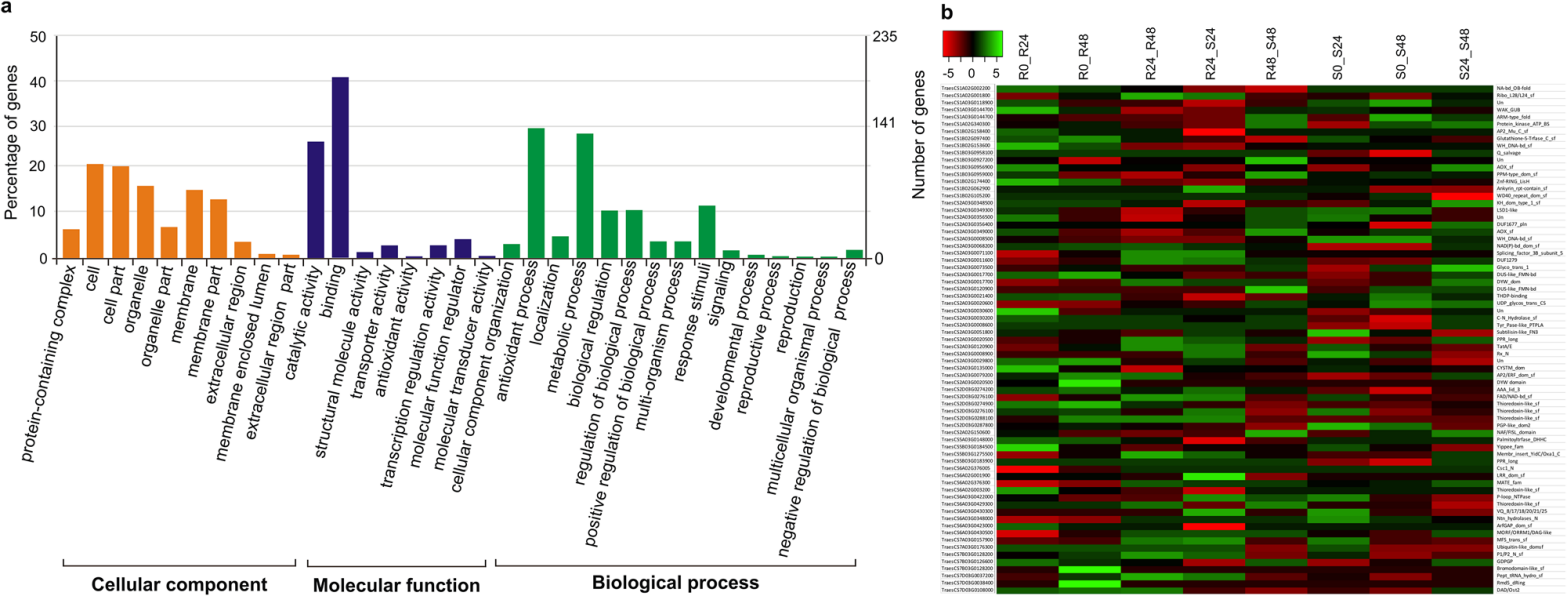
(**a**) Gene ontology (GO) for differentially expressed candidate genes (DECGs) identified in the present study; (**b**) Heat maps exhibiting 71 DECGs that encoded important proteins and are associated with the MQTLs. The scale shown on the top of left of the heat map shows the fold change value that varies in the heat-maps proteins encoded by DECGs.

*Gene Ontology (GO) of CGs*. The gene ontology (GO) of CGs suggested occurrence of GO terms belonging to all three widely known classes of functions, including cellular, molecular, and biological processes. The details of these functions are presented in Fig. 3a.

*In Silico expression of CGs*. The in silico expression analysis for 411 CGs led to the identification of only 71 differentially expressed genes (DECGs). The comparison of expression involved either between the two (R = Resistant or S = Susceptible) at different durations or between different genotypes (R vs. S) at the same durations after inoculation. Thus, the following eight comparisons of expression were available: (i) R0 vs. R24; (ii) R0 vs. R48; (iii) R24 vs. R48; (iv) R24 vs. S24; (v) R48 vs. S48; (vi) S0 vs. S24; (vii) S0 vs. S48; and (viii) S24 vs. S48 (See Fig. 3b).

The fold change of the DECGs ranged from 5.4 fold upregulation to -5.0 fold downregulation. The maximum difference in expression of DECGs was observed in the pair of susceptible and resistant genotypes at 24 hpi and 48 hpi (R24 vs. S24 and R48 vs. S48). In these comparisons, four DECGs were significantly downregulated in four MQTLs (MQTL1, MQTL2, MQTL9, and MQTL15). Similarly, nine DECGs were upregulated in six MQTLs (MQTL3, MQTL4, MQTL5, MQTL6, MQTL12, and MQTL13). In both comparisons, sample taken from a resistant genotype was used as the control, suggesting that more genes are upregulated in S genotype relative to those, which are upregulated in R genotypes. It also suggested that resistance is achieved by silencing many genes that are responsible for susceptibility.

An the above analysis involving comparison of R and S genotypes, DECGs also led to the identification of 58 unique domains in which 10 protein domains were associated with biotic stress resistance. Among the downregulated DECGs, the encoded proteins belonged to following four main categories: (i) Nucleic acid-binding, (ii) RING-type zinc-finger, (iii) Palmitoyltransferase, and (iv) GDP-L-galactose/GDP-D-glucose phosphorylase. Similarly, in upregulated DECGs, the following six protein domain were identified: (i) PPM-type phosphatase domain superfamily, (ii) DUS-like, FMN-binding domain, (iii) AOX protein family, (iv) Thioredoxin-like superfamily, (v) NB-LRR domain superfamily, and (vi) VQ domain (Fig. 3b, Supplementary Table S6).

In comparison of expression within the same genotype (either compatible interaction of incompatible interaction) at different intervals, some DECGs showed distinct expression patterns associated with compatible interactions (S0 vs. S24, S0 vs. S48, and S24 vs. S48), while others were known to be associated with incompatible interactions (R0 vs. R24, R0 vs. R48 and R24 vs. R48) (Supplementary Table S6). The functional analysis revealed distinct trends in protein domain functions between the two reaction types. In the compatible response, genes with downregulated expression were enriched for the following domains: (i) NAD(P) binding, (ii) carbon–nitrogen metabolism, (iii) specific regions of PRORP proteins, (iv) protein degradation (ubiquitin-like), and (v) protein synthesis (peptidyl-tRNA hydrolysis). In contrast, in the upregulated response involved in incompatible interactions, following domains were involved: (i) phosphoglycolate phosphatase-like domain, potentially linked to phosphate metabolism; (ii) splicing factor 3B subunit 5 and (iii) CYSTM protein domains. These domains suggested potential role of RNA processing and transmembrane signaling in defense response (Fig. 3b, Supplementary Table S6).

Biological pathways were also identified in all the 71 DECGs using Kyoto Encyclopedia of Genes and Genomes (KEGG) pathway analysis (KEGG database utilised with permission). Four significantly enriched KEGG pathways induced by spot blotch were identified; these pathways included the following: (i) basal transcription factors (BSFs), (ii) glutathione metabolism, (iii) ribosome processing and (iv) protein processing in endoplasmic reticulum (ER). These four pathways were associated with the following four DECGs i.e. TraesCS1B02G153600 (MQTL3), TraesCS1B02G097400 (MQTL2), TraesCS1A02G001800 (MQTL1), and TraesCS6A02G003200 (MQTL12).

## Discussion

In recent years, molecular breeding involving the use of DNA markers for MAS has become routine in conventional plant breeding. This is particularly true, when improvement of quantitative traits is involved. However, for MAS, robust markers that are suitable to be used for MAS are not always available. In such studies, MQTLs derived from QTLs identified in earlier studies may sometimes prove useful. For spot blotch and related traits, the present meta-QTL study is the first of its kind. The study utilized all known QTLs not only for spot blotch resistance but also those associated with five other related traits, which often accompany spot blotch resistance (for identity of five traits, see above).

As reported in results, in the present study, using 228 QTLs that were available from published literature, only 84.0 (36.8%) could be projected onto the consensus map, because only these had the complete information needed for MQTL analysis. This gave 31 MQTLs, of which 15 MQTLs were based each on a single QTL and were therefore rejected, thus leaving only 16 MQTLs derived from 69 QTLs. The remaining 144 QTLs could not be projected due to one of the following reasons: (i) there were no flanking markers that the original and consensus maps shared, and (ii) the CIs were relatively large^19^. Notably, SG trait was the only trait besides spot blotch resistance that was directly targeted in QTL mapping within the context of spot blotch^23^. In previous studies^24–28^, the remaining traits (except BPR), namely GLAD, GLNS, FLS, were generally also subjected to mapping of tolerance against abiotic stress factors in wheat. A 14.2 -fold reduction in the number of genomic areas or QTLs linked to spot blotch resistance in wheat was achieved through the identification of 16 MQTLs from 228 QTLs. This projection success percentage is less than that of previous studies on MQTL analysis conducted for different wheat disease resistances, where the following projection rates were available: 44.0, 60.62, 66.6, and 75.2%^15,16,19^. The present study also adds to the examples of MT-MQTLs involving disease resistance, because 12 of the 16 MQTLs were MT-MQTLs. Earlier MQTL studies in wheat have been carried out for a variety of traits, including tolerance against abiotic and biotic stresses. For instance, Liu et al.^14^ identified 20 MQTLs for tan spot using 106 QTLs, Amo et al.^17^ reported 35 MQTLs for leaf rust using 128 QTLs, and Jan et al.^15^ discovered 61 MQTLs for stripe rust using 184 QTLs. MQTLs for multiple disease resistance (MDR) were also identified in two of our own studies (see later for some details).

Although our focus in the present study was primarily on SBR, a five other traits known to be associated with SBR were also included. Among these associated traits,, SG trait was of special interest, since it has the unique characteristic of delaying senescence in leaves and other plant parts, enabling them to maintain high chlorophyll levels and sustain photosynthetic activity for an extended period. The association of SG with spot blotch has also been reported in wheat, where a significant negative correlation (0.73) between SG and AUDPC (a measure of spot blotch severity) has been reported^23,37^. In a number of studies, SG has often been used as a selection criterion for tolerance against abiotic stresses also, including heat and drought^16,24–26,28,38^. Thus, this adaptive trait (SG) is also particularly useful in adverse environments, since it facilitates an improved grain-filling process^27^. The genotypes that are characteristics of SG and SBR also exhibit tolerance/resistance against heat/drought, suggesting a shared physiological mechanism for response to abiotic and biotic stresses including SBR^39^.

Since six different traits were used in the present study, it was also possible to identify MT-MQTL, which can be used for improvement of more than one traits using the same MQTL (see later in Discussion). In the published literature, only two other studies from our own work were available, which involved identification of MT-MQTLs, both involving resistance against a number of diseases. In one study, Saini et al.^19^ reported MQTLs for MDR involving five unrelated diseases (STB, SNB, FHB, KB, and LS), while Pal et al.^20^ reported MDR MQTLs for only the three rusts, including leaf rust, stem rust, and stripe rust. In the present study, out of 16 MQTLs, 12 involved more than one trait and were therefore described as MT-MQTL (involving 2–4 traits); no MT-MQTL was available for 5 or 6 traits (Table 1). Among these 12 MT-MQTLs, on the basis of priority, three MT-MQTL are being recommended for MAS involving SBR with other associated traits (see later).

The merit of MQTLs relative to the corresponding QTLs used for MQTL analysis, depends on four key attributes, including PVE, LOD score. CI and the number of QTLs involved in each individual MQTL. Following are some details of comparison of these four attributes in MQTL and QTLs used for MQTL analysis, suggesting that MQTL showed an improvement over QTLs: (i) The PVE of the MQTLs ranged from 1.6 to 55.2% (with a mean of 13.6%), while that of the QTLs ranged from 5 to 10% in 72 QTLs, followed by 10–15% in 60 QTLs, and 15 to 20% in 39 QTLs. This suggested that PVE% improved in MQTLs. (ii) The LOD scores of MQTLs ranged from 3.0 to 11.0; in contrast, in QTLs, the LOD score ranged from 2 to 4 in 83 QTLs, from 4 to 6 in 75 QTLs, and 6–8 in 48 QTLs, suggesting that the LOD score also improved in MQTL. (iii) The CI in MQTLs largely ranged from 1.5 to 20.7 cM (except five MQTLs, where it ranged from 36.1 to 57.8 cM) as against 0.62–272 cM for the QTL used for MQTL analysis, suggesting a major reduction in the length of the CI, thus making these MQTL relatively more robust.(iv) Similarly, 8 MQTLs were each based on two QTLs, and MQTL3 was based on 17 QTLs, suggesting that the number of QTLs associated with individual MQTLs is large (at least in some MQTLs), thus improving the credibility of the utility of MQTLs (Table 1).

The results of the present MQTL study on SB in wheat can also be compared with those available for several other wheat diseases, including three rusts^9,15,16,18^, fusarium head blight (FHB)^10^ and tan spot^14^. Following are the some details about the MQTLs for disease resistance identified in other studies: (i) The PVE ranged from 4.7 to 51.0% for three rusts, 6.2–22.3% for FHB, and 6.3–27.0% for tan spot^14^. (ii) The LOD score for MQTL ranged from 3.0 to 28.0 for three rusts^20^, from 1.0 to 62.0 for FHB^12^, and from 3.9 to 17.0 for tan spot^14^. (iii) The CI in these earlier studies ranged from 0.04 to 83.5 cM for three rusts^20^, < 10.0–95.0 cM for FHB^10^ and 0.9–8.1 cM for tan spot^14^. For MT-MQTLs for MDR in earlier studies, the PVE ranged from 2.2% to 51.2%, the LOD score ranged from 2.9 to 48.4 and the CI ranged from 0.04 to 15.2 cM^19^.

For validation, a study of colocalization of MQTLs identified in the present study with MTAs identified in published literature was also undertaken. For this purpose, MTAs were available for only SBR, SG, and BPR; for other traits, no GWAS were available. For these three traits, 13 (> 81.0) of the 16 MQTs were colocalized with GWAS-MTAs (Supplementary Table S4. These results can be compared with similar validation studies undertaken earlier. In these earlier studies, co-localization ranged from 38.7 to 90.5%^17,20,40–44^. The high rate of colocalization suggests the presence of causal polymorphisms in MQTLs identified in the present study.

Another component of the present study was the identification of CGs associated with MQTLs. The distribution of CGs ranged from none in MQTL7 to a maximum of 157 in MQTL2, with an average of ~ 33 CGs per MQTL (Supplementary Table S5). This data suggests a potential role for these CGs in spot blotch resistance, warranting further investigation. The R domains in proteins encoded by CGs are known to be involved in plant defense mechanisms, strengthening the hypothesis of their association with disease resistance. In wheat, similar studies on CGs associated with MQTLs were earlier conducted for traits like fusarium head blight^12^, stripe rust tolerance^15,16^, leaf rust^17^, tan spot resistance^14^, MDR^19,20^.

Among a large number of CGs identified to be associated with MQTLs in the present study, 71 DECGs were available, which encode proteins carrying domains with known roles in plant defense. Some of these genes were downregulated (FC < − 2.0), while others were upregulated, when a resistant genotype was compared with a susceptible genotype at two different durations after inoculation (R24 vs. S24 and R48 vs. S48). The protein domains involved in downregulation included the following: nucleic acid-binding domain, RING-type zinc-finger domain, and palmitoyltransferase domain. Similarly, the upregulated genes encoded proteins carrying the following domains: AOX protein family, AOX protein family, NBS-LRR and VQ protein. These results are also in agreement with the results of several earlier studies conducted either in Arabidopsis or in wheat^45–52^, suggesting that these DECGs deserve further detailed study.

In the present study, we also validated 13 (> 81.0%) of the 16 MQTLs using the previously conducted GWAS. This also allowed selection of the following for MQTLs based on high PVE, LOD value, and lower CIs: MQTL3, MQTL4, MQTL5, and MQTL8. Genomic selection models can also utilize these markers to enhance the accuracy of resistance prediction. On the basis of present study, we also recommend further research to clone and functionally characterize the identified candidate genes (CGs). These genes have the potential to be used to improve wheat resistance but require validation through techniques like gene cloning, reverse genetics, or omics approaches.

In the present study, among the four R genes (*Sb1* to *Sb4*) known for spot blotch, *Sb1* is the only Sb gene, which colocalize with one of the 16 MQTLs, namely MQTL16, suggesting that the list of MQTLs identified is certainly not exhaustive and exclusive and that there must be more SB MQTLs (associated with *Sb2*, *Sb3* and *Sb4*) to be identified in future.

Among the 12 MT-MQTLs also, three MT-MQTLs (MQTL3, MQTL5 and MQTL8) are recommended for MAS on the basis of criteria outlined above for selection of MQTLs. MQTL16 carrying the R gene *Sb1* is also recommended for MAS. Only future QTL and MQTL studies will allow identification of MQTLs associated with the remaining R genes, namely *Sb1*, *Sb3* and *Sb4*, Some of the DECGs identified during the present study may also be utilized for further studies.

## Summary

In the present meta-QTL study, 228 QTLs were available from earlier QTL interval mapping studies, involving spot blotch and five related traits. Only 69 QTL could be projected leading to the identification of 16 MQTLs; 12 of these MQTLs were MT-QTLs, each involving 2–4 traits, suggesting that the same meta-QTL may be used for improvement of more than one trait. Superiority of the MQTLs over corresponding QTLs was demonstrated on the basis of improved PVE%, LOD scores and reduced length of CI^11^. As many as 13 of the 16 MQTs were colocalized with GWAS-MTAs, thus placing s higher level of confidence in these MQTLs. A large number of CGs were also identified, one of them differentially expressed. The integration of desirable alleles from MQTL3, MQTL4, MQTL5, and MQTL8 can provide high and stable spot blotch resistance. MQTL3, with 17 initial QTLs for four traits including spot blotch disease resistance QTLs, may be particularly useful in developing spot blotch-resistant cultivars through MAS. MQTL5 and MQTL8 each have eight initial QTLs. All five of these MQTLs meet the preferred criteria mentioned earlier and can be easily transferred into susceptible genotypes using MAS. Other MQTLs may also confer resistance against spot blotch disease after introgression into susceptible wheat backgrounds.

## Material and methods

### Literature survey for spot blotch related QTLs

For the identification of MQTLs, 228 QTLs were available from 21 different studies, with 24 mapping populations related to spot blotch resistance (SBR) and five other related traits. The related traits included SG, FLS, GLAD, GLMS, and BPR. GLAD and GLMS are both related traits, the former representing duration and the latter representing the extent of green leaf area; these have been treated as separate traits in published literature and therefore retained as two different traits in the present study also (Supplementary Table S1).

### Development of consensus map

A consensus map was developed using 72,788 markers, including SNPs, DArT, SSRs, and EST-based markers, that were available in five distinct genetic linkage maps^53–57^. The software Lp merge was used for developing the consensus map^58^. Similar consensus maps were earlier developed and utilized to find the MQTLs for different traits in wheat^15,59^. The QTLs and the flanking markers for different traits were also used in the preparation of the consensus map.

### QTL projection and MQTL analysis

Out of 228 QTLs, only 84 had all the information necessary for MQTL projection. These QTLs were used for projection, and meta-analysis was conducted using BioMercator V4.2^60^. When a marker's genetic position was uncertain, the QTL was projected on the consensus map, and the markers closest to the QTL were accepted as associated markers. Wherever the CI of an individual QTL was not available (there were 5 QTLs for which CIs were not available), the CI (95%) was estimated utilizing the following two equations, where N is the size of the population: (i) CI = 163 ÷ (N × R^2^) for the QTLs identified using RIL populations^61^, (ii) CI = 530 ÷ (N × R^2^) for F_2_ and backcross (BC) populations^62^, and (iii) CI = 287 ÷ (N × R^2^) for the QTLs detected using doubled haploid (DH) population^9^.

QTLs with inadequate information (with no PVE values, or no LOD scores or no information on genetic position, etc.) were eliminated. Following two different methods were used during the analysis, depending upon the available number of QTLs: (i) When the number of QTLs per linkage group was not more than 10, the method proposed by Goffinet and Gerber^63^ was used; in this approach, we tested all possible combinations based on the Akaike Information Criterion (AIC) to determine the number of underlying meta-QTLs that best fit the available results, and (ii) when the number of projected QTLs per linkage group was > 10, the two-step analysis proposed by Veyrieras et al.^64^ was employed; this is the best fit meta-QTL model based on the following parameters: (i) AIC, (ii) corrected AIC, (iii) corrected AIC with a penalty factor of 3, (iv) Bayesian Information Criterion (BIC), and (v) approximate weight of evidence criterion (AWE). The statistical algorithms and techniques furnished in the software are provided by Sosnowski et al.^65^.

### Validation of MQTLs with GWAS-based MTAs

MQTLs identified in the present study were validated with MTAs reported from 21 independent GWA studies, including 11 GWA studies on SBR, 6 on SG and 4 on BPR. No GWAS studies were found for other traits used in the present study. These GWA studies utilized populations of hexaploid wheat only (e.g., spring and winter wheat), with population sizes ranging from 101^66^ (BPR) to 1384^27^ (SG), and phenotyped at one or more locations across five different countries (India, Mexico, Pakistan, USA for SBR, and China for BPR). The details of population size, associated diseases, genotyping platform, number of markers used, and MTAs detected in these studies are available in Table 3. The physical positions of each significant and stable marker associated with the trait were obtained from the respective studies or the JBrowse WHEAT URGI database (https://urgi.versailles.inra.fr/jbrowseiwgsc/) and CerealsDB (https://www.cerealsdb.uk.net/cerealgenomics/CerealsDB/indexNEW.php). Subsequently, the physical positions of these MTAs were compared with the physical coordinates of the MQTLs; MQTL co-localizing each with at least one MTA was considered a GWAS-validated/verified MQTL (Supplementary Table S5).

**Table 3:** Summary of genome-wide association studies (GWAS) in wheat on spot blotch (SBR), black point (BPR) and stay green (SG) used in the present study.

Type of wheat (Panel type)	Genotyping platform/Number MTA’s	No of MTAs/QTLs	Model	Country	References
SBR					
566 wheat accessions	832 DArT markers	41	MLM	USA	Adhikari et al.^67^
528 hexaploid spring wheat accessions	5634 markers of wheat 90 K iSelect SNP chip	6	GLM	USA	Gurung et al.^68^
287 WAMI spring wheat panel	iSelect 90 K SNP (21,132)	6	MLM	India	Ahirwar et al.^69^
159 wheat lines derived from CIMMYT	87,096 GBS-SNP markers	96	MLM	Pakistan	Jamil et al.^70^
294 genotypes of HWWAMP	15,590 Illumina iSelect 90 K SNP markers	8	GLM, MLM, CMLM, ECML, FaST-LMM, and SUPER	USA	Ayana et al.^71^
139 advanced breeding lines of spring wheat from CIMMYT	14,063 GBS-SNP markers	29	FarmCPU and MLM	India	Tomar et al.^72^
289 WAMI spring wheat panel	13,589 Illumina iSelect 90 K SNP markers	25	MLM	India	Singh et al.^73^
301 wheat genotypes representing a collection from Afghanistan	8425 DArT markers	35	GLM, MLM, MLMM, and FarmCPU	India	Bainsla et al.^74^
1,092 advanced breeding lines from	6736 DArTseq markers	24	MLM	India, Mexico	Juliana et al.^75^
438 SHW lines, generated by the CIMMYT	5800 DArT P/L markers	11	MLM	Mexico	Lozano-Ramirez et al.^76^
303 Spring Wheat Reference Set (SWRS), collected from CIMMYT	12,196 SNP markers based on DArT-seq	89	CMLM, SUPER, MLMM, FarmCPU, BLINK	India	Singh et al.^77^
BPR					
166 diverse cultivars of China	90 K Iselect SNP chip (81,587 SNPs) and Affymetrix 660 K SNP, (630,517 SNPs)	26	MLM, FarmCPU	China	Liu et al.^78^
101 wheat genotypes of China	50 K SNP arrays (53,063 SNPs)	23	MLM	China	Li et al.^66^
Two wheat panels. Panel I consisted of 163 common wheat cultivars and Panel II consisted of 243 common wheat cultivars or advanced breeding lines	Panels I and II were genotyped using the wheat 90 K and 660 K SNP arrays, respectively	27	MLM	China	Lv et al.^25^
272 Chinese wheat landraces	660 K SNP arrays	11	MLM	China	Tang et al.^79^
SG					
PNW winter wheat population (*n* = 402) assembled from advanced breeding lines, cultivars, and parent lines	6492 SNP markers	19	MLM	USA	Gizaw et al.^80^
A total of 322 wheat genotypes	29,949 SNP markers	74	FarmCPU	USA	Ward et al.^81^
A germplasm panel consisted of 70 bread wheat accessions obtained from Egypt, Syria and Iran	Five SSRs and 93 SNP marker	1	GAPIT	Egypt	Alsamman et al.^82^
1384 lines of NAM mapping population	18,827 SNP markers	22	MLM	Australia	Christopher et al.^27^
A set included 160 (Nebraska Duplicate Nursery—NDN) lines	16,835 SNP markers	28	GLM, MLM and FarmCPU	Egypt	Sallam et al.^83^
A set of 685 spring wheat genotypes was randomly selected from a MR-NAM	15,146 SNP markers	40	MLM	Germany	Vukasovic et al.^84^

### Candidate genes (CGs)

For identification of CGs, the sequences of flanking markers of MQTLs (obtained from GrainGenes; https://wheat.pw.usda.gov/GG3; and CerealsDB) were BLASTed (maximum E-value = 1E−100, minimum 95 percent sequence identity) against the wheat reference genome (IWGSC RefSeq v2.1) to obtain the physical positions of these flanking markers. The physical positions of the array-based markers were collected from the JBrowse wheat genome browser (https://wheat-urgi.versailles.inra.fr/Tools/JBrowse). The 2 Mb physical region (one Mb on either side) around the peaks of MQTLs was searched for the CGs^19^ using the BioMart (https://plants.ensembl.org/biomart/martview/3730aa7b8d0c2bccb6248a87470a97df) tool of Ensembl Plants. We estimated the expression of CGs using our own transcriptome data from spot blotch disease at three time points, i.e., 0 h, 24hpi and 48hpi. An online tool, WEGO (wego.genomics.org.cn/), accessed June 25, 2022, was used to graphically represent the results of proteins related to CGs.

### Expression analysis

For the expression analysis of DECGs, transcriptome data generated using spot blotch infested leaf samples from both resistant (Yangmai6) and susceptible (Sonalika) genotypes were collected at three time points, i.e. 0 h, 24 hpi and 48hpi. Using the transcriptomic data of both genotypes, a total eight pairs of comparisons (R0 vs. R24, R0 vs. R48, R24 vs. R48, R24 vs. R48, R24 vs. S24, R48 vs. S48, S0 vs. S24, S0 vs. S48, and S24 vs. S48) were made. Differential expression was recorded as a fold change against the control (resistant). The transcriptomic data for other traits was not available. The heat map for DECGs was developed using an online tool called heat-mapper^35^ (http://www.heatmapper.ca/expression). Identification of main biological pathways significantly enriched with differentially expressed genes (DEGs) were identified using the database Kyoto Encyclopedia of Genes and Genomes (KEGG)^85^. Significance of these pathways were determined using adjusted P-values.

### Human and animal rights

The research involved in the manuscript did not involve live vertebrates, higher invertebrates, or human subjects. Ethical considerations related to these elements are not applicable to this study.

### Supplementary Information

Supplementary Information 1.

Supplementary Information 2.

Supplementary Information 3.

Supplementary Information 4.

Supplementary Information 5.

Supplementary Information 6.

## Data Availability

All data generated or analysed during this study are included in this published article and its supplementary information files.

## Code availability

Publicly available softwares were used in the study.
